# Downregulation of NAGLU in VEC Increases Abnormal Accumulation of Lysosomes and Represents a Predictive Biomarker in Early Atherosclerosis

**DOI:** 10.3389/fcell.2021.797047

**Published:** 2022-01-26

**Authors:** Changchang Xing, Zhongyi Jiang, Yi Wang

**Affiliations:** ^1^ Department of Cardiology, Shanghai General Hospital, Shanghai Jiao Tong University School of Medicine, Shanghai, China; ^2^ Department of General Surgery, Shanghai General Hospital, Shanghai Jiao Tong University School of Medicine, Shanghai, China

**Keywords:** early atherosclerosis, NAGLU, vascular endothelial cell, VEGFR2, ERK, lysosome, bioinformatics analysis

## Abstract

Cardiovascular diseases (CVDs), predominantly caused by atherosclerosis (AS), are the leading cause of mortality worldwide. Although a great number of previous studies have attempted to reveal the molecular mechanism of AS, the underlying mechanism has not been fully elucidated. The aberrant expression profiling of vascular endothelial cells (VECs) gene in early atherosclerosis (EAS) was analyzed according to the dataset (GSE132651) downloaded from the Gene Expression Omnibus (GEO) database. We primarily performed functional annotation analysis on the downregulated genes (DRGs). We further identified that α-N-acetylglucosaminidase (NAGLU), one of the DRGs, played a critical role in the progression of EAS. NAGLU is a key enzyme for the degradation of heparan sulfate (HS), and its deficiency could cause lysosomal accumulation and lead to dysfunctions of VECs. We found that siRNA knockdown of NAGLU in human umbilical vein endothelial cell (HUVEC) aggravated the abnormal accumulation of lysosomes and HS. In addition, the expression of NAGLU was reduced in the EAS model constructed by *ApoE* ^−/-^ mice. Furthermore, we also showed that heparin-binding EGF-like growth factor (HB-EGF) protein was upregulated while NAGLU knockdown in HUVEC could specifically bind to vascular endothelial growth factor receptor 2 (VEGFR2) and promote its phosphorylation, ultimately activating the phosphorylation levels of extracellular signal-regulated kinases (ERKs). However, the application of selective VEGFR2 and ERKs inhibitors, SU5614 and PD98059, respectively, could reverse the abnormal lysosomal storage caused by NAGLU knockdown. These results indicated that downregulation of NAGLU in HUVEC increases the abnormal accumulation of lysosomes and may be a potential biomarker for the diagnosis of EAS.

## Introduction

Cardiovascular diseases (CVDs), predominantly caused by atherosclerosis (AS), are the leading cause of morbidity and mortality worldwide (Herrington et al., 2016). AS is a chronic progressive inflammatory disease and a progressive pathological process with the buildup of intimal plaque in the artery wall (Paiva-Lopes and Delgado Alves, 2017). The pathogenesis of AS, indeed, is complicated and involves multiple cell types, including vascular endothelial cells (VECs), smooth muscle cells, and macrophages (Higashi et al., 2016). Previous research has shown that dysfunction of VECs (e.g., endothelial cell activation) that line the arterial vasculature is an important contributor to the pathobiology of AS (Gimbrone and García-Cardeña, 2016), which is also the earliest detectable change in the progression of AS (Stary, 2000; Virmani et al., 2000). Furthermore, dysfunction of VECs can also induce these cells to produce and secrete cytokines and chemokines, leading to monocyte/lymphocyte recruitment and infiltration into the subendothelium (Hansson, 2005), which further aggravates the progression of AS plaque formation (Tedgui and Mallat, 2006; Bosisio et al., 2014; Paramel Varghese et al., 2016). Therefore, exploring detectable biomarkers for the progression of AS and investigating the potential mechanisms of atherosclerotic plaque formation are pivotal for the prevention and treatment of AS.

Over the years, numerous bioinformatics tools have leveraged increasingly abundant genomic data to facilitate the discovery of potential diagnostic and therapeutic targets for AS (Herrington et al., 2018; Meng et al., 2019). In light of this, our study utilized a dataset downloaded from the Gene Expression Omnibus (GEO) database to analyze the aberrant expression profile of dysfunctional VEC genes in EAS. We then focused on the downregulated genes (DRGs) and performed functional annotations, including Gene Ontology (GO), Kyoto Encyclopedia of Genes and Genomes (KEGG) enrichment analysis, and protein–protein interaction (PPI) network, to reveal the molecular mechanism of the development and progression of EAS. It is noteworthy that α-N-acetylglucosaminidase (NAGLU), which is one of the DRGs, plays a critical role in the dysfunction of VECs in EAS.

NAGLU is a key enzyme required for the degradation of glycosaminoglycans (GAGs) heparan sulfate (HS) (De Pasquale and Pavone, 2019). Deficiency of NAGLU mainly causes lysosomal accumulation and urinary excretion of HS, which is characteristic of mucopolysaccharidosis type IIIB (MPS-IIIB) (Roy et al., 2012; Prill et al., 2019). Of note, the aberrant accumulation of HS is not only in the lysosome of the cell but also in the cell surface and extracellular matrix (Pan et al., 2005; Watson et al., 2014). During the development and progression of atherosclerosis, HS associated proteoglycans (HSPGs) play an important role in binding to low-density lipoproteins (LDL), which facilitates the formation of atherosclerotic plaques (Madonna and De Caterina, 2014; Tsiantoulas et al., 2021). Furthermore, the previous study has also implicated that the alterations in GAGs metabolism are involved in the pathological processes of atherosclerotic vasculature (Nakashima et al., 2008). However, many questions remain about understanding the underlying mechanisms of dysfunction of VECs in EAS.

Based on bioinformatics analysis, the present study used small interfering RNA (siRNA) to generate a cellular model of NAGLU knockdown in HUVEC for further verification. Downregulation of NAGLU increased the abnormal accumulation of lysosomes and HS in HUVEC, which was associated with the activation of vascular endothelial growth factor receptor 2(VEGFR2). Hence, we investigated the VEGFR2 signaling pathway in HUVEC with NAGLU knockdown accelerating the pathological changes of cells, which provides new insight into the diagnostic biomarkers and molecular mechanisms of EAS.

## Materials and Methods

### Data Source

The gene expression profiling dataset GSE132651 (Hebbel et al., 2020) was obtained from the GEO database (GPL96 platform [HG-U133A], Affymetrix Human Genome U133A Array) (Wang et al., 2016), which included six subjects with normal endothelial function and 13 subjects with abnormal endothelial function associated with EAS.

### Data Standardization and Repeatability Testing

Statistical data analysis and visualizations were generated using the R programming language. First, the Robust Multi-array Average (RMA) algorithm was performed to preprocess and normalize the raw data from the GSE132651 dataset using R. Then, Pearson’s correlation test (PCT) was used to evaluate the correlation of biological repeats between all samples and visualize the results with a heatmap through R. Principal component analysis (PCA) was performed using R to reduce the dimension of the phenotypic dataset and obtain a cluster map of the samples.

### Construction of Weighted Gene Co-Expression Network Analysis (WGCNA)

WGCNA analysis was performed on the normalized data using the R package “*WGCNA*” (Langfelder and Horvath, 2008). WGCNA analysis provides new biological insight to describe the correlation patterns among different samples, which generates undirected networks to identify modules of highly correlated genes and to calculate the membership measures of different modules. The main procedure of WGCNA consists of the following parts: 1) identification of soft power; 2) identification of modules by hierarchical clustering and Dynamic Tree Cut; 3) constructing a gene co-expression network; 4) studying module relationships through Eigengene Networks; and 5) calculation of module and character association.

### Identification of Aberrant Expression Profiling

The aberrant gene expression profiling was analyzed and visualized by volcano maps using the R packages “*GEOquery*” (Davis and Meltzer, 2007), “*limma,*” and “*ggplot2*.” Considering that the difference in gene expression profiling between EAS and the normal group is relatively insignificant, we set the thresholds as *p* < 0.01 and fold change ≥0.4 in this study to explore more genes related to the progression of EAS. Moreover, heatmaps of aberrant gene expression profiling in the normal and abnormal endothelial function groups (NG and ANG, respectively) were drawn using the R package “*pheatmap*.”

### Functional Annotations Including GO, KEGG, and Metascape Analyses

GO enrichment analysis is currently the most commonly used ontology application in bioinformatics and provides a dynamically changing standard vocabulary to describe functional aspects of gene products, which covers three aspects of biology, including biological process (BP), cellular component (CC), and molecular function (MF). KEGG is a database resource for understanding the high-level functions and utilities of biological systems. Functional annotations were performed by GO and KEGG through the R package “*clusterProfiler*” (Yu et al., 2012), and items with *p.adjust* <0.05 were further visualized. Metascape (https://metascape.org/gp/index.html#/main/step1) is also an online database for gene annotation and analysis, including GO and KEGG analyses (Zhou et al., 2019). Terms with a *p*-value < 0.01 and an enrichment factor >1.5 were collected and grouped into clusters based on their membership similarities.

### PPI Enrichment Analysis

The construction of the PPI network for DRGs was carried out with the STRING database (https://string-db.org/) (Szklarczyk et al., 2019). Proteins with an interaction score > 0.4 will be further visualized with Cytoscape (version 3.8.2) software for the PPI network (Smoot et al., 2011). Then, the Molecular Complex Detection algorithm (MCODE, version 2.0.0) was utilized to cluster a given network based on the topology to find densely connected areas (Bader and Hogue, 2003). The analysis parameters of MCODE were set as Degree Cutoff = 2, K-Core = 2, Node Score Cutoff = 0.2, and Max. Depth = 100. The gene set with the highest cluster score will be selected for further analysis.

### Multiple Gene Set Enrichment Analysis (GSEA) of Single Gene

The GSEA algorithm was used to determine the alteration of multiple signaling pathways related to gene expressions (Subramanian et al., 2005). The number of permutations was set as 1,000, and functional enrichment analyses were performed to generate significant gene sets correlated with the expression levels of NAGLU based on the Molecular Signatures Database (MSigDB). All gene sets with nominal *p* value < 0.05 were considered to be statistically significant and selected for visualization using R.

### Antibodies and Chemicals

Primary antibodies: rabbit anti-NAGLU/NAG monoclonal antibody (mAb) (ab214671), rabbit anti-VEGFR2 mAb (ab134191), and rabbit anti-ERK1+ERK2 mAb (ab184699) (Abcam, Cambridge, MA, United States); rabbit anti-phospho-VEGFR2 (Tyr1175) mAb (#2478) and rabbit anti-phospho-p44/42 mitogen-activated protein kinases (MAPK) (Erk1/2) (Thr202/Tyr204) mAb (#4370) (Cell Signaling Technology, Leiden, the Netherlands); mouse anti-CD31/PECAM-1 (H-3) mAb (sc-376764) (Santa Cruz Biotechnology, Heidelberg, Germany); rabbit anti-TUBB polyclonal antibody (D223070) (Sangon Biotech, Shanghai, China).

Secondary antibodies: HRP-conjugated goat anti-rabbit IgG (D110058) and HRP-conjugated goat anti-mouse IgG (D110098) (Sangon Biotech); anti-mouse IgG (H + L), F (ab')2 Fragment (Alexa Fluor^®^ 488 Conjugate) (#4408) and anti-rabbit IgG (H + L), F (ab')2 Fragment (Alexa Fluor^®^ 594 Conjugate) (#8889) (Cell Signaling Technology).

Chemicals: DAPI Fluoromount-G™ (36308ES20, Yeasen, Shanghai, China); 7.5 and 10% SDS-PAGE Reagent kits (EpiZyme, Shanghai, China); polyvinylidene fluoride (PVDF) membranes (Millipore, Billerica, MA, United States); BCA Protein Quantification Kit, LysoTracker Red (C1046) and Triton X-100 (P0096) (Beyotime, Shanghai, China); Radio Immunoprecipitation Assay (RIPA) lysis buffer (Sangon Biotech); fetal bovine serum (FBS) (GIBCO, Karlsruhe, Germany); lectin from *Triticum vulgaris* (wheat) FITC conjugate (L4895, Sigma-Aldrich, St. Louis, MO, United States); SU5614 (S0278) and PD98059 (S1177) (Selleck, Shanghai, China); FastPure^®^ Cell/Tissue Total RNA Isolation Kit V2 (RC112-01) and Taq Pro Universal SYBR qPCR Master Mix (Q712-02) (Vazyme Biotech, Nanjing, China); PrimeScript™ RT Reagent Kit with gDNA Eraser (Perfect Real Time) (RR047A, Takara, Beijing, China); Lipofectamine 2000 reagent (Invitrogen, Carlsbad, CA, United States).

### Cell Culture

HUVEC (a cell line derived from CRL-1730, ATCC, Manassas, VA, United States) was cultured in modified low glucose DMEM (Gibco, Thermo Fisher Scientific, Waltham, MA, United States) supplemented with 10% FBS (Gibco) and 1% penicillin-streptomycin (Gibco) in 5% CO_2_ at 37°C.

### Cell Transfection

The siRNA targeting NAGLU (si-NAGLU) and the negative control sequence (si-NC) were synthesized by GenePharma (GenePharma, Shanghai, China). The target sequences for si-NAGLU-8 and si-NAGLU-6 were 5′-GGC​CAC​UUU​AAC​UGU​UCC​UTT-3′ and 5′-GGC​ACA​UCA​AGC​AGC​UUU​ATT-3′, respectively. The target sequence for si-NC was 5′-UUC​UCC​GAA​CGU​GUC​ACG​UTT-3′. HUVEC was transfected with siRNA using Lipofectamine 2000 according to the manufacturer’s protocol.

### Quantitative Real-Time Polymerase Chain Reaction (qRT-PCR)

Total RNA was extracted from the HUVECs using an Isolation Kit following the manufacturer’s instructions. Synthesis of cDNA was conducted using a reverse transcription kit with 1,000 ng RNA. qRT-PCR was performed with the SYBR qPCR Master Mix kit. The primer sequences used for gene analysis were as follows: NAGLU-Forward: 5′-CAA​CAG​GTA​CCG​CTA​TTA​CC-3′, NAGLU-Reverse: 5′-GCA​GGA​CCA​GTA​AAG​AAC​TC-3′; β-actin-Forward: 5′-GTG​GGG​CGC​CCC​AGG​CAC​CA-3′, β-actin-Reverse: 5′-CTC​CTT​AAG​TCA​CGC​ACG​ATT​TC-3′. The cycling procedure started at 95°C for 30 s followed by 40 cycles of 95°C for 10 s and 60°C for 30 s, and the last step was 72°C for 20 min. Relative mRNA expression was calculated using the 2^−ΔΔCt^ method.

### Western Blotting

Total protein extraction of HUVECs was carried out using RIPA lysis buffer and quantified by a BCA kit. Protein lysates were separated through SDS-PAGE and subsequently transferred onto PVDF membranes. Membranes were blocked with 5% bovine serum albumin (BSA) for 1 h at room temperature (RT) and then incubated with primary antibodies overnight at 4°C. After washing with Tris Buffered Saline with Tween^®^20 (TBST), membranes were incubated with secondary antibodies for 1 h at RT. Finally, the protein signals were detected by enhanced chemiluminescence (Beyotime).

### Fluorescent Probe Detection

LysoTracker staining has been commonly used for labeling lysosomes. In summary, the LysoTracker reagent was diluted at a ratio of 1:15,000 and preheated at 37°C for 10 min. Then, the cell culture medium was removed, and the diluent was added and incubated for 30 min at 37°C. After washing, fresh culture medium was added and subsequently observed under a confocal fluorescence microscope.

Lectin is a fluorescent probe that can specifically label HS. HUVECs were cultured on the round coverslips. After removing the cell culture medium and washing with PBS, 4% PFA was used to fix the cells for 30 min. Then, the cells were washed with PBS 3 times, permeabilized with 0.1% Triton X-100 for 10 min at RT, and incubated with 50 μg/ml lectin-FITC diluted in PBS for 1 h at RT. Finally, after washing 3 times with PBS, the glass slides were turned upside down on glass slides containing the Anti-Fade Mounting Medium (E675011, Sangon Biotech) and observed under the confocal fluorescence microscope.

### Animal Studies

Eight-week-old male *ApoE* ^*−/−*^ mice (C57BL/6) were obtained from Cavens Lab Animal (Changzhou, China) and used to construct the EAS model. Those mice were randomized into a control group fed a normal chow diet (NCD, TD08485, Harlan Teklad) (*n* = 5) and an EAS group fed a high-fat diet (HFD, TD02028, Harlan Teklad) (*n* = 5). All mice were sacrificed after 4 weeks of feeding for further analysis. Due to the difficulty of operation, the number of models successfully constructed and the samples obtained are as follows: NCD = 3, HFD = 4. The successful construction of the EAS animal model was verified by oil red O staining (E607319-0010, Sangon Biotech) on part of the arterial tissues. All animal experiments were approved by the Institutional Animal Care and Use Committee of Shanghai General Hospital.

### Immunofluorescence Staining

Briefly, aortic arch samples from *ApoE* ^*−/−*^ mice were fixed with 4% PFA and then embedded in paraffin. After drying, dewaxing, antigen retrieval, and blocking treatments, four-micron-thick sections were incubated with primary antibodies (CD31, 1:100; NAGLU, 1:100) overnight at 4°C in a humidified box. After washing, the sections were incubated with secondary antibodies at RT for 1 h and protected from light, followed by DAPI Fluoromount-G™ at RT for 10 min. Finally, the images were captured with a fluorescence microscope.

### Statistical Analysis

The results were shown as the mean ± SD. The two-tailed Student’s *t* test was used for comparisons between the two groups. The Pearson correlation test was used for correlation analysis between samples. All statistical analyses were performed with GraphPad Prism 9 (GraphPad Software, La Jolla, CA, United States), and the R programming algorithms were performed using R software 4.1.1. A *p* value < 0.05 was considered statistically significant.

## Results

### Validation and Processing of the Datasets

**FIGURE 1:**
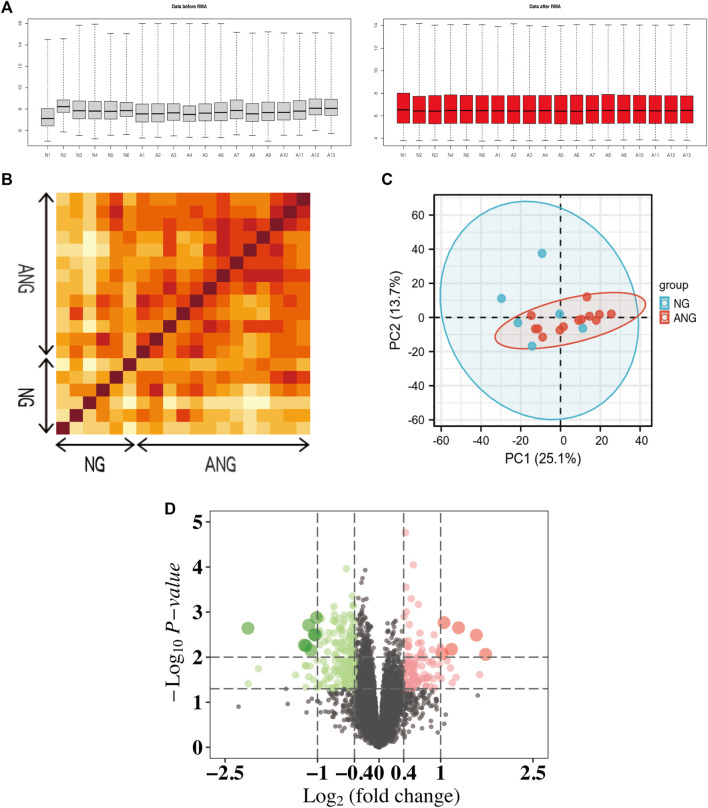
Normalization and repeatability analysis of the dataset. **(A)** RMA algorithm normalization of samples from the GSE132651 dataset. Left: data before RMA; right: data after RMA. **(B)** Pearson’s correlation analysis of NG and ANG samples from the GSE132651 dataset. The color reflects the intensity of the correlation, where the darker the color, the stronger the correlation intensity. **(C)** PCA of samples from the GSE132651 dataset. Each point represents a sample in the figure, and the distance between the two samples represents the difference in gene expression patterns of the two samples. **(D)** The volcano plot illustrates the differences between NG and ANG samples after analysis of the GSE132651 dataset using R.

To allow a comprehensive analysis of microarray data, we performed the RMA algorithm, a form of quantile normalization, to normalize the dataset (Figure 1A). We then followed PCT and PCA dimension reduction to further validate the interpretability and scalability of the data. According to the results of the PCT, we showed that there was a strong correlation between the respective samples of the NG or ANG in the GSE132651 dataset (Figure 1B). The quality and reliability between samples of the two groups then underwent dimension reduction by using PCA, where the dimension of principal component-1 (PC1) demonstrated the close association between them (Figure 1C). Additionally, the volcano plot visualized the aberrant gene expression profiling in the GSE132651 dataset (Figure 1D).

### Identification of Co-Expression Modules Using WGCNA

**FIGURE 2:**
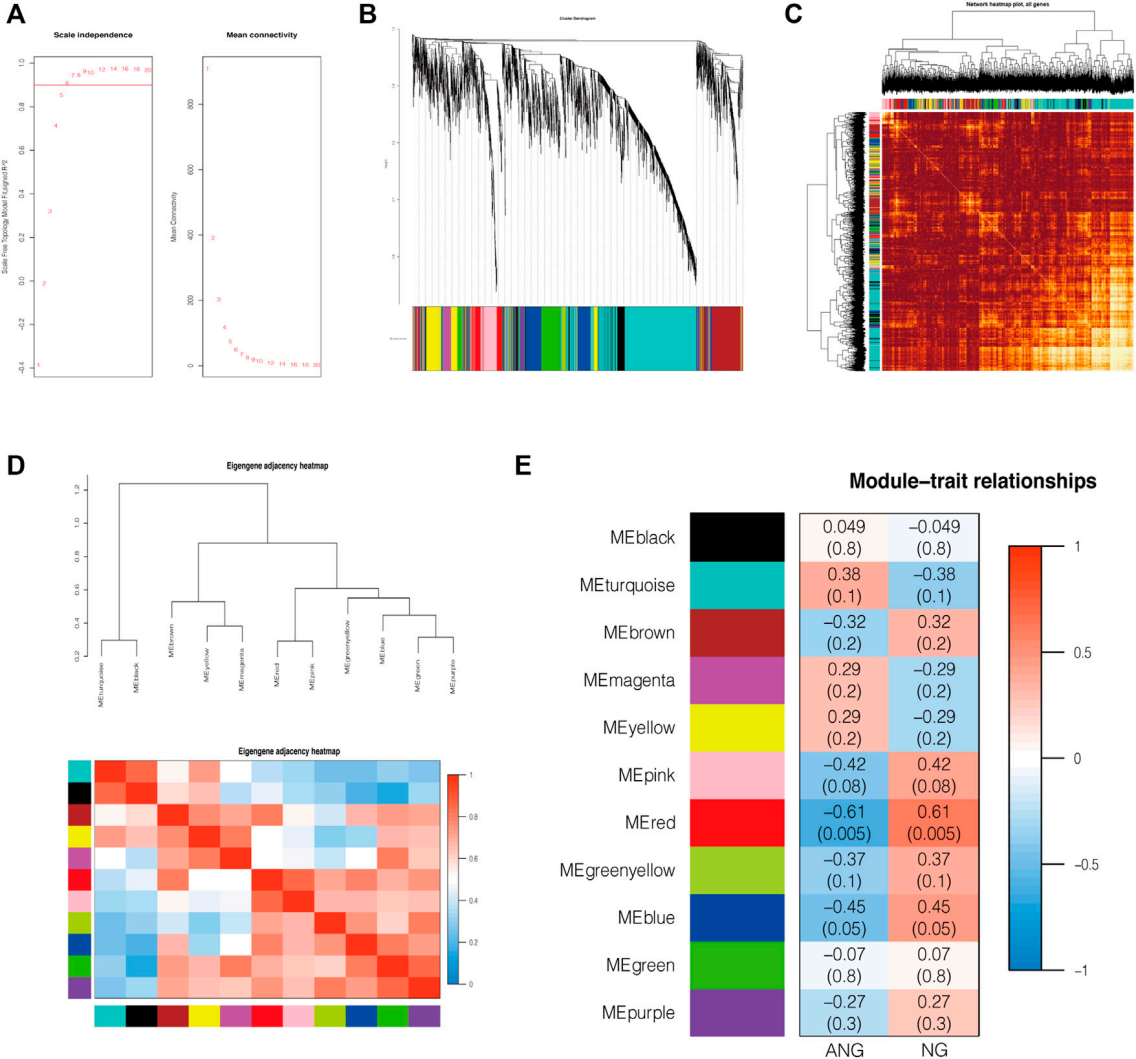
Construction of co-expression modules by WGCNA. **(A)** The relatively balanced scale independence and mean connectivity in Scale-free topology analysis. The power value was 6, which was the lowest power for the scale-free topology fit index of 0.9. **(B)** Construction of co-expression modules by the WGCNA package through R. Genes are represented by branches, and co-expression modules are shown in different colors below in figure. **(C)** Network heatmap of the interaction relationship of different modules. **(D)** Eigengene adjacency heatmap plot of different modules. **(E)** Heatmap of the correlation between module eigengenes and the disease status of EAS. The red module was the most negatively correlated with status. NG: normal endothelial function groups; ANG: abnormal endothelial function groups.

To identify whether the data met the characteristics of the scale-free network, we selected 0.9 as the scale-free topology fit index for network topology analysis and determined relatively balanced scale independence and mean connectivity (Figure 2A). We then set 0.25 as the mergeCutHeight to merge similar modules and generated 11 important modules (Figure 2B). As shown in the network heatmap plot, there was no significant difference in the interaction between different modules, which further demonstrated the high scale independence among the modules (Figure 2C). Moreover, the eigengene adjacency heatmap plot also showed the mutual independence between each module (Figure 2D). The red module was the most negatively correlated with the status of EAS, whereas it was the most positively correlated with the status of normal status (Figure 2E).

### Functional Annotations (GO and KEGG Analyses) and PPI Network of Gene Expression Profiling

**FIGURE 3:**
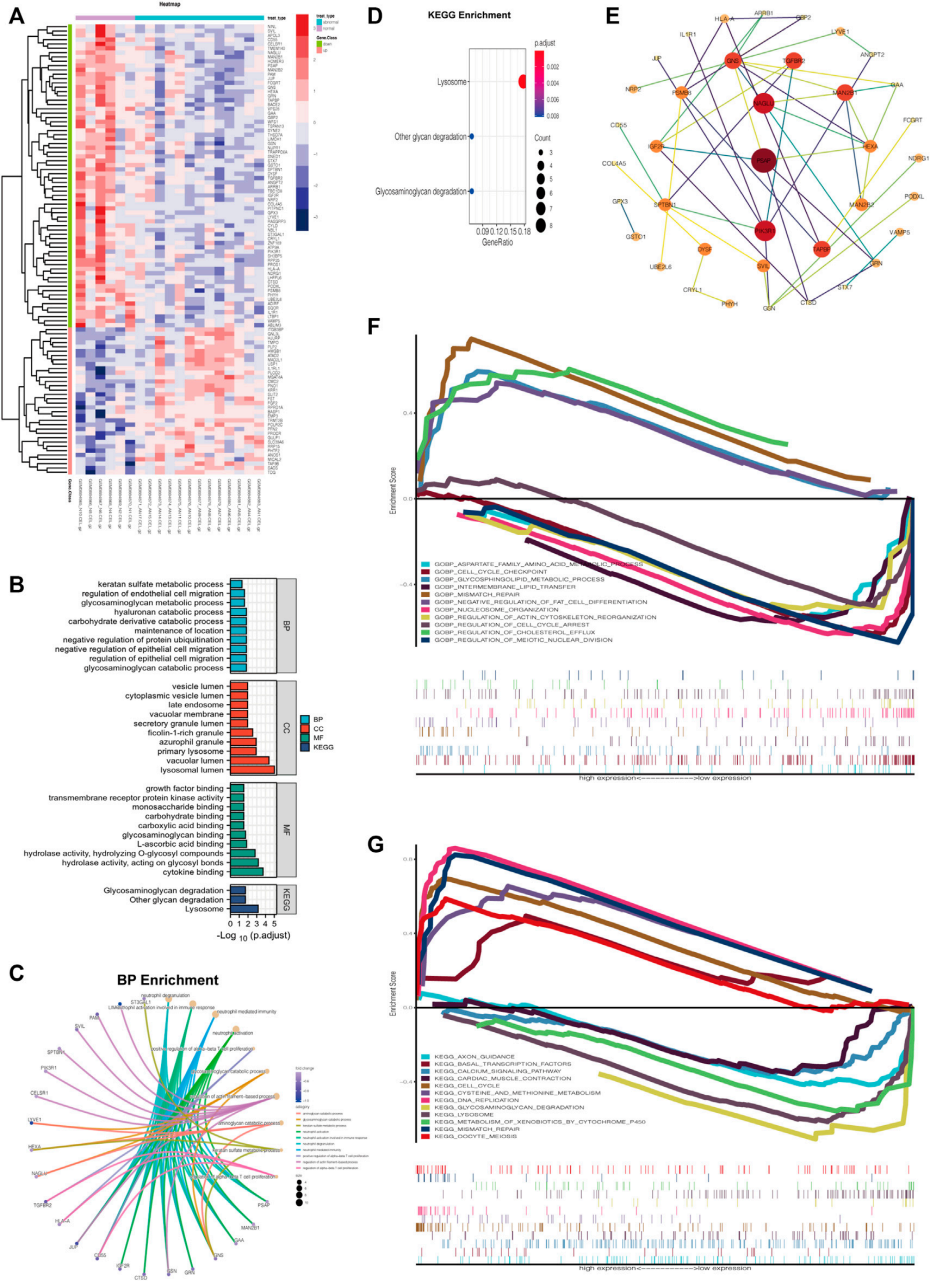
Aberrant gene expression profiling and functional annotation enrichment analyses. **(A)** Heatmap of aberrant gene expression profiling. **(B)** GO (including BP, CC, and MF) and KEGG analyses of aberrant gene expression profiling. **(C)** GO BP enrichment of DRGs using R. **(D)** KEGG pathways enrichment of DRGs using R. **(E)** Construction of the PPI network of DRGs *via* Cytoscape software. **(F)** Multiple GSEA of varied expression of NAGLU using GO BP analysis (c5). **(G)** Multiple GSEA of varied expression of NAGLU using KEGG analysis (c2).

A heatmap was generated to visualize the differential gene expression matrix between NG and ANG (Figure 3A). GO analysis revealed that the primary variations in BP included GAGs catabolic process and regulation of epithelial and endothelial migration; the results of CC were mainly enriched in lysosomal lumen and vacuolar lumen; cytokine binding, GAG binding, and growth factor binding were primarily enriched in MF (Figure 3B). Analysis of the KEGG pathway indicated that all aberrantly expressed genes were mainly enriched in lysosomes, GAGs, and other glycan degradation (Figure 3B). In addition, a similar enrichment analysis was performed through Metascape, and the results were mainly enriched for cytokine binding, lysosomal lumen, and GAGs catabolic process (Supplementary Figure S1). It is noteworthy that we further found that the DRGs in ANG were the main regulators of the GO functional enrichment (Figure 3C, Supplementary Figures S2A,B) and KEGG pathways with the highest enrichment scores (Figure 3D). On this basis, the Cytoscape software was utilized to construct the PPI network of DRGs, which showed the degree distribution of all selected genes (Figure 3E).

### Identify NAGLU as a Key Regulator in EAS

The result of MCODE analysis identified the top 5 hub genes in the PPI network of DRGs, including NAGLU, GAA, MAN2B1, HEXA, and GNS (Table 1). Among them, NAGLU, ranked first based on the MCODE score, is a known critical regulator of GAGs degradation and lysosome signaling pathways (Supplementary Figures S2C,D). Furthermore, Gene Set Enrichment Analysis (GSEA), which uses the Molecular Signatures Database gene sets as the reference gene sets, was performed using c2 (c2. cp.kegg.v7.4. symbols.gmt) and c5 (c5. go.bp.v7.4. symbols.gmt). It was suggested that varied expression of NAGLU was markedly related to the aspartate family amino acid metabolic process and glycosphingolipid metabolic process (Figure 3F). Citing c2 collection to perform the KEGG analysis, it was indicated that lysosome and GAGs degradation signaling pathways were enriched under low expression levels of NAGLU (Figure 3G).

**TABLE 1:** Identification of the top 5 hub genes of DRGs in the PPI network using MCODE analysis.

Name	logFC	MCODE score	*p*-value
NAGLU	−0.437106083	3	0.002107708
GAA	−0.491661026	3	0.003726139
HEXA	−0.456769835	2.7	0.005265312
MAN2B1	−0.451152751	2.7	0.004458614
GNS	−0.584413576	2.7	0.001801124

### NAGLU Knockdown Induces Lysosomal Defects in HUVEC and Low Expression of NAGLU in the EAS Animal Model

**FIGURE 4:**
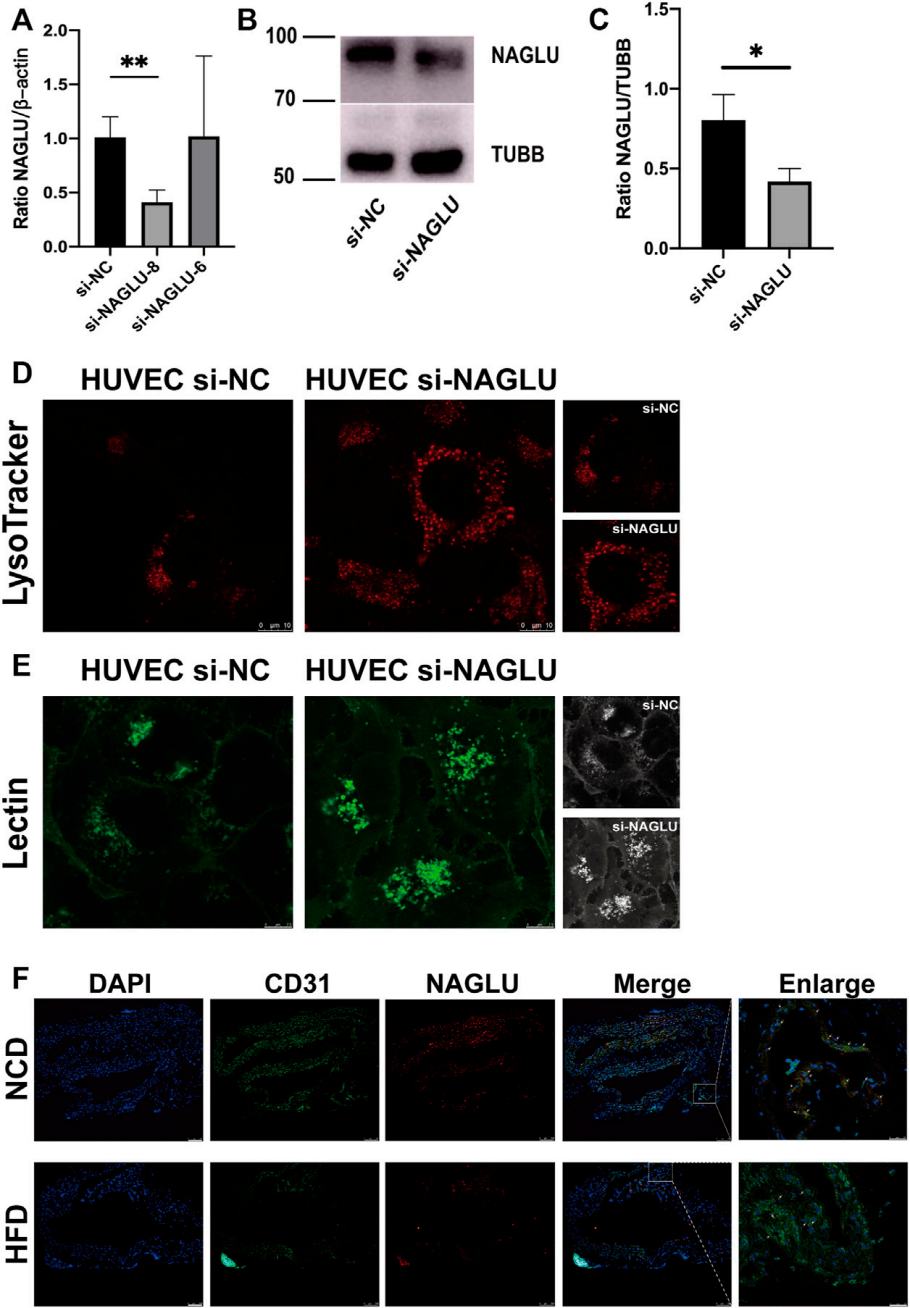
NAGLU reduction in the EAS animal model and NAGLU knockdown in HUVEC result in defective lysosomal storage. **(A)** The mRNA expression levels of NAGLU in HUVEC after transfection by qRT-PCR. si-NAGLU-8 was used for subsequent research. **(B,C)** The protein expression levels of NAGLU in HUVEC after transfection with si-NAGLU and si-NC as measured by Western blotting; TUBB was used as a control. **(D)** Representative images of lysosomes labeled with the LysoTracker probe in HUVEC si-NAGLU and HUVEC si-NC. Scale bars: 10 μm. **(E)** Representative images of HS labeled with lectin probe in HUVEC si-NAGLU and HUVEC si-NC. Scale bar: 7.5 μm. **(F)** Representative images of *ApoE* ^*−/−*^ mice aortic arches stained with DAPI, NAGLU, and CD31. NCD: normal chow diet; HFD: high-fat diet. *p < 0.05, **p < 0.01, Student’s t-test.

To explore the molecular mechanism of NAGLU expression in EAS, we generated a cellular model by knockdown of the gene expression of NAGLU in HUVEC through siRNA. The mRNA expression level of NAGLU was significantly reduced under si-NAGLU-8 knockdown compared to si-NC (Figure 4A). Similar results were obtained at the protein expression level through si-NAGLU-8 knockdown (Figures 4B,C). Since NAGLU is involved in the regulation of lysosome and GAGs degradation signaling pathways, we investigated whether NAGLU knockdown could induce aberrant accumulations of lysosomes and HS in endothelial cells. Compared with HUVEC si-NC, HUVEC si-NAGLU showed more substantial lysosomal accumulation by LysoTracker probe stain detection (Figure 4D). In addition, based on the specific recognition of HS by fluorescent lectin, we found that there were stronger and more widely distributed lectin signals in HUVEC si-NAGLU than in HUVEC si-NC (Figure 4E). In the EAS animal model constructed with *ApoE* ^*−/−*^ mice, staining with oil red O revealed increased lipid accumulation in the arterial endothelium of the HFD group compared with the NCD group, which proved the successful construction of the EAS animal model (Supplementary Figure S3). We then observed that the expression of NAGLU was significantly reduced in the endothelial cells of the aortic arch of the HFD group compared to the mice fed an NCD (Figure 4F).

### NAGLU Knockdown Upregulates HB-EGF and Promotes VEGFR2/ERK Activations in HUVEC

**FIGURE 5:**
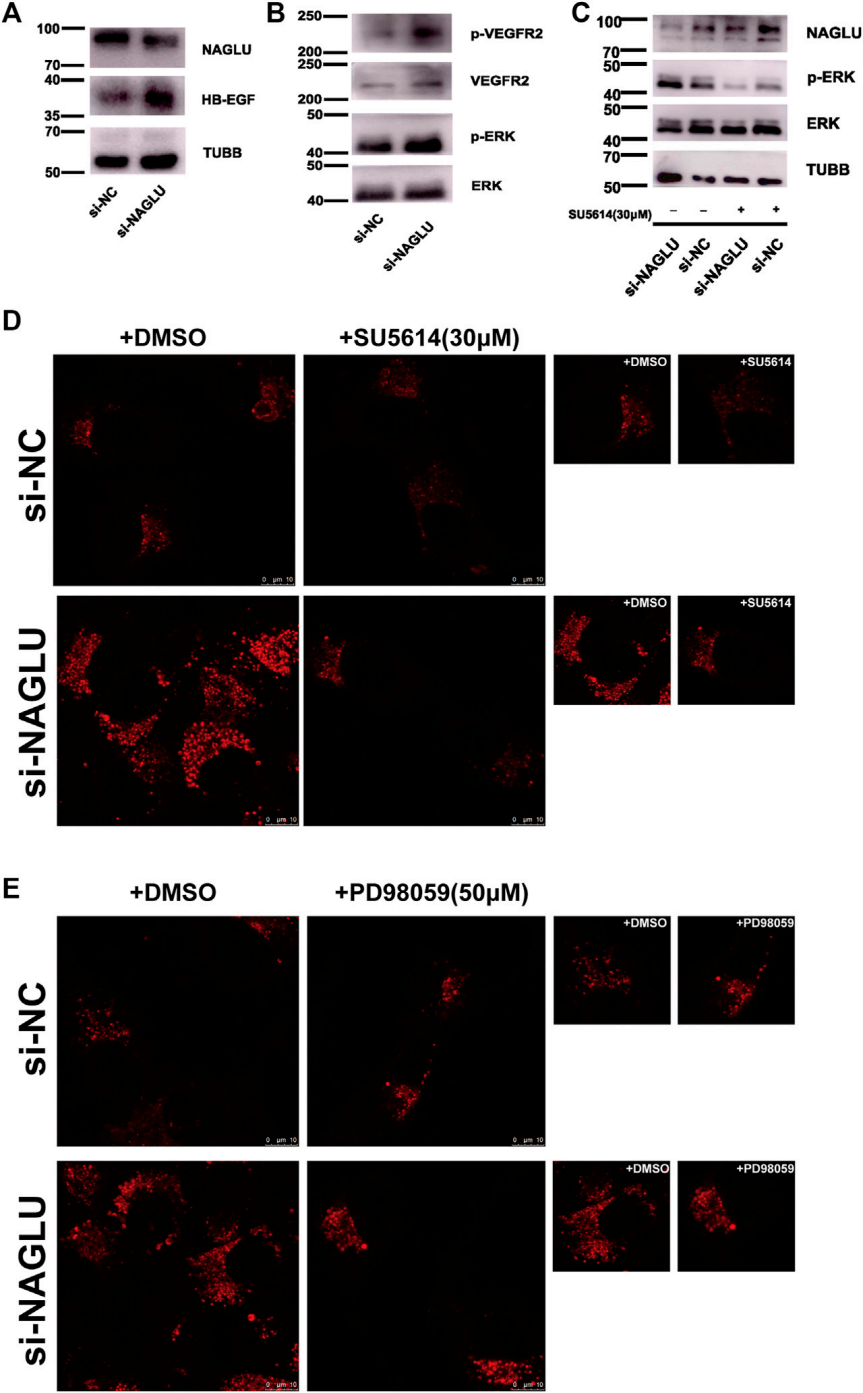
NAGLU knockdown causes aberrantly lysosomal accumulation in HUVEC targeting VEGFR2/ERKs and promotes EAS. **(A)** The protein expression levels of HB-EGF in HUVEC after transfection with si-NAGLU and si-NC as measured by Western blotting. TUBB was used as a control. **(B)** VEGFR2 and ERKs phosphorylation levels in HUVEC si-NAGLU and HUVEC si-NC were measured by Western blot. To monitor the equal loading of protein in the gel lanes, the upper blot was stripped and tested using anti-VEGFR2 and anti-ERKs antibodies, respectively. **(C)** VEGFR2 inhibition reduces ERK1/2 phosphorylation levels in HUVEC si-NAGLU. HUVEC si-NAGLU and HUVEC si-NC were both treated or untreated with 30 μM VEGFR2 inhibitor SU5614 for 12 h and then measured by Western blot. **(D)** VEGFR2 inhibition reduces aberrantly lysosomal accumulation in HUVEC si-NAGLU. Representative images of lysosomes labeled by the LysoTracker probe in HUVEC si-NAGLU and HUVEC si-NC, both treated with 30 μM SU5614 for 12 h. Scale bars: 10 μm. **(E)** MAPK/ERKs inhibition reduces aberrant lysosomal accumulation in HUVEC si-NAGLU. Representative images of the lysosomes labeled by the LysoTracker probe in HUVEC si-NAGLU and HUVEC si-NC, both treated or untreated with 50 μM PD98059 for 24 h. Scale bars: 10 μm.

Western blot analysis was used to study the molecular mechanism of lysosomal accumulation caused by NAGLU knockdown in HUVEC. Previous studies have shown that extracellular HSPGs can bind and regulate the activity of HB-EGF (Iwamoto et al., 2010), whereas HB-EGF can induce VEGF production in several diseases (Nakai et al., 2009; Karakida et al., 2011; Shim et al., 2016). In addition, the VEGF–VEGFR2 pathway has been implicated in atherosclerosis (Khurana et al., 2005; Ylä-Herttuala et al., 2007; Taher et al., 2016). We then verified that HB-EGF was remarkably upregulated in HUVEC si-NAGLU compared with HUVEC si-NC (Figure 5A **)**, which further bound to VEGFR2 and promoted the phosphorylation levels of VEGFR2 (Figure 5B). Moreover, VEGFA/VEGFR2, upstream of the MAPK/ERK signaling pathway, has been confirmed to facilitate intraplaque neovascularization in atherosclerosis (Hu et al., 2018). Consistent with these findings, we also demonstrated that the activation of VEGFR2 could enhance ERK phosphorylation (Figure 5B). Treatment with the specific VEGFR2 inhibitor SU5614 (Sharifpanah et al., 2015) significantly reduced the phosphorylation levels of ERK in HUVEC si-NAGLU compared to those in the untreated group (Figure 5C). Additionally, we evaluated whether treatment with SU5614 affects the accumulation of lysosomes in HUVEC. Staining with the LysoTracker probe showed that SU5614 treatment obviously reduced lysosomal accumulation in HUVEC si-NAGLU, whereas it did not affect lysosomal compartments in HUVEC si-NC (Figure 5D). Furthermore, we investigated whether HUVEC si-NAGLU treatment with PD98059 (De Pasquale et al., 2018), a selective inhibitor of the MAPK/ERK pathway, would also reverse the phenomenon of lysosomal storage. As expected, the accumulation of lysosomes in PD98059-treated HUVEC si-NAGLU was significantly reduced, whereas it did not affect HUVEC si-NC (Figure 5E).

## Discussion

Atherosclerosis is known to be a disease of chronic inflammation and a common underlying cause of cardiovascular morbidity and mortality worldwide. It is worth noting that endothelial cell dysfunction (ECD) in arterial vasculature lesions is the earliest detectable change in a variety of pathological processes leading to atherosclerotic lesions (Stary, 2000; Virmani et al., 2000). However, the mechanism of ECD in EAS is not fully understood.

To provide new insight into the potential pathogenesis of EAS, our study analyzed a microarray dataset of aberrant gene expression profiling of VECs in EAS. After normalization, we verified the repeatability of the data and the independence between the datasets, and a total of 104 varied genes were identified between NG and ANG. Functional annotation enrichment analysis, including GO and KEGG analyses, found that these genes were significantly related to lysosome and GAGs degradation. To explore the critical factors that regulate the lysosome and GAGs degradation pathways, a subanalysis of the upregulated and downregulated genes in the ANG showed that downregulated genes were significantly related to these pathways. The following PPI network and MCODE algorithm of Cytoscape further demonstrated that NAGLU, a known pivotal regulator in regulating lysosome and GAGs degradation, may play a critical role in the progression of EAS.

Previous studies have confirmed that NAGLU deficiency disrupts the lysosomal turnover of HS, leading to abnormal accumulation of lysosomes and HS in MPS IIIB (De Pasquale et al., 2018; Prill et al., 2019). Furthermore, myocardial hypertrophy, as one of the clinical manifestations of MPS IIIB, is closely related to the abnormal accumulation of lysosomes caused by NAGLU deficiency, which contributes to early mortality (De Pasquale et al., 2018). Moreover, early studies showed significantly impaired endothelial function in MPS patients with GAG metabolism defects, which were more likely to develop EAS (Brosius and Roberts, 1981; Yano et al., 2014). Consistently, we confirmed the reduction of NAGLU expression in the EAS model constructed using *ApoE* ^*−/−*^ mice. In addition, abnormal accumulation of lysosomes and HS was also observed in HUVEC si-NAGLU. The results of these phenotypes confirm that bioinformatics analysis is helpful for research on disease progression.

The ECD in EAS is characterized by the infiltration of lipoprotein in the subendothelial space, which can trigger a series of complex pathogenesis, including the activation of endothelial cells and generating growth factors and chemokines, the recruitment and transformation of macrophages into foam cells, the proliferation and migration of smooth muscle cells and synthesis of extracellular matrix, the lipid retention caused by interaction between lipoproteins and GAGs, and finally the formation of fibromuscular plaques (Ross, 1999; Pirillo et al., 2013; Hultgårdh-Nilsson et al., 2015; Gimbrone and García-Cardeña, 2016). In this regard, we were mainly concerned about the regulatory mechanism of defective lysosomal clearance in EAS caused by GAGs metabolism defects.

It has been proved that the extracellular HSPGs accumulation contributes to GAGs metabolism defects (Pan et al., 2005), which could bind and regulate the activity of HB-EGF (Iwamoto et al., 2010; De Pasquale et al., 2018). Even cells treated with HB-EGF *in vitro* could induce the production of VEGF (Nakai et al., 2009; Shim et al., 2016). In addition, a growing body of evidence suggests that activation of the VEGF–VEGFR2 pathway is involved in the GAGs metabolism (Zhang, 2010; Freudenberg et al., 2015) and the development of AS (Khurana et al., 2005; Ylä-Herttuala et al., 2007; Taher et al., 2016). Along this line, we demonstrated that the expression levels of HB-EGF were upregulated in HUVEC si-NAGLU, as well as the specific activation of VEGFR2. Moreover, we revealed that the application of VEGFR2 inhibitor can reverse the abnormal levels of lysosomal accumulation in HUVEC si-NAGLU, which indicated the regulatory role of VEGFR2 in VECs in the pathogenesis of EAS. We hypothesized that the increased phosphorylation of VEGFR2 in NAGLU knockdown HUVEC by lysosomal defects could be credited to the interaction with the heparin-like domain of cell surface HSPGs (Dougher et al., 1997; Ashikari-Hada et al., 2005). In fact, the phosphorylation levels of VEGFR2 could be reduced by depletion of cell surface HS due to heparinase treatment (Ashikari-Hada et al., 2005). These findings suggest that further studies are needed to elaborate the specific signaling pathway of VEGFR2 activation caused by abnormal HS accumulation in VECs in EAS.

The key molecular players involved in the progression of ECD and EAS include platelet endothelial cell adhesion molecule-1 (PECAM-1), VE-cadherin, VEGFRs, MAPK/ERK, signal transducer and activator of transcription-3 (STAT-3), nuclear factor kappa B (NF-κB), PI3K/AKT, eNOS, KLF-4, and KLF-2 (Morris et al., 2020). Remarkably, neovascularization in atherosclerotic lesions plays a critical role in plaque growth and instability, and numerous studies have shown that the MAPK/ERK pathway regulated by VEGFR2 participates in angiogenesis (Koch and Claesson-Welsh, 2012; Claesson-Welsh, 2016; Camaré et al., 2017). Herein, we proved that NAGLU knockdown promoted ERKs activation in HUVEC, whereas this phenomenon could be inhibited by the VEGFR2 inhibitor. Furthermore, either inhibiting the phosphorylation of VEGFR2 or ERKs could reduce the abnormal accumulation of lysosomes in HUVEC si-NAGLU. These results indicated that upregulation of HBEGF promoted the phosphorylation of VEGFR2 and further activated the ERK pathway in HUVEC, which may explain the mechanism by which defects in lysosomal storage and GAGs degradation aggravated EAS.

There were two major limitations of our research. On the one hand, although we showed a significantly defective lysosomal storage phenotype of NAGLU and the pathway it targets, the exact mechanism remains unclear. NAGLU, almost an unknown protein in the progression of EAS, needs further deeper investigation. On the other hand, we hope that NAGLU could be verified in clinical samples, but limited by the difficulty of sample acquisition, it has not been realized.

In conclusion, we elaborated on the pivotal role of NAGLU in GAGs degradation and lysosomal storage in EAS, wherein it targets the VEGFR2/ERK pathway. Furthermore, NAGLU may represent a predictive biomarker for ECD in EAS.

## Data Availability

The original contributions presented in the study are included in the article/Supplementary Material. Further inquiries can be directed to the corresponding author.

## Abbreviations

NAGLU, α-N-acetylglucosaminidase; CVDs, cardiovascular diseases; AS, atherosclerosis; EAS, early atherosclerosis; ECD, endothelial cell dysfunction; VECs, vascular endothelial cells; GEO, Gene Expression Omnibus; DRGs, downregulated genes; HS, heparan sulfate; HUVEC, human umbilical vein endothelial cell; HB-EGF, heparin-binding EGF-like growth factor; GAGs, glycosaminoglycans; HSPGs, HS associated proteoglycans; VEGFR2, vascular endothelial growth factor receptor 2; ERKs, extracellular signal-regulated kinases; GO, gene ontology; KEGG, Kyoto Encyclopedia of Genes and Genomes; PPI, protein–protein interaction; MPS, mucopolysaccharidosis; HFD, high-fat diet; NCD, normal chow diet; NG, normal endothelial function groups; ANG, abnormal endothelial function groups.
