# Supplementary material for: Downregulation of NAGLU in VEC Increases Abnormal Accumulation of Lysosomes and Represents a Predictive Biomarker in Early Atherosclerosis
Source: Front Cell Dev Biol. 2022 Jan 26;9:797047. doi: 10.3389/fcell.2021.797047 (PMC8826576; doi:10.3389/fcell.2021.797047)
Supplement: Supplementary file 2 [file DataSheet1.docx]

Supplementary Material
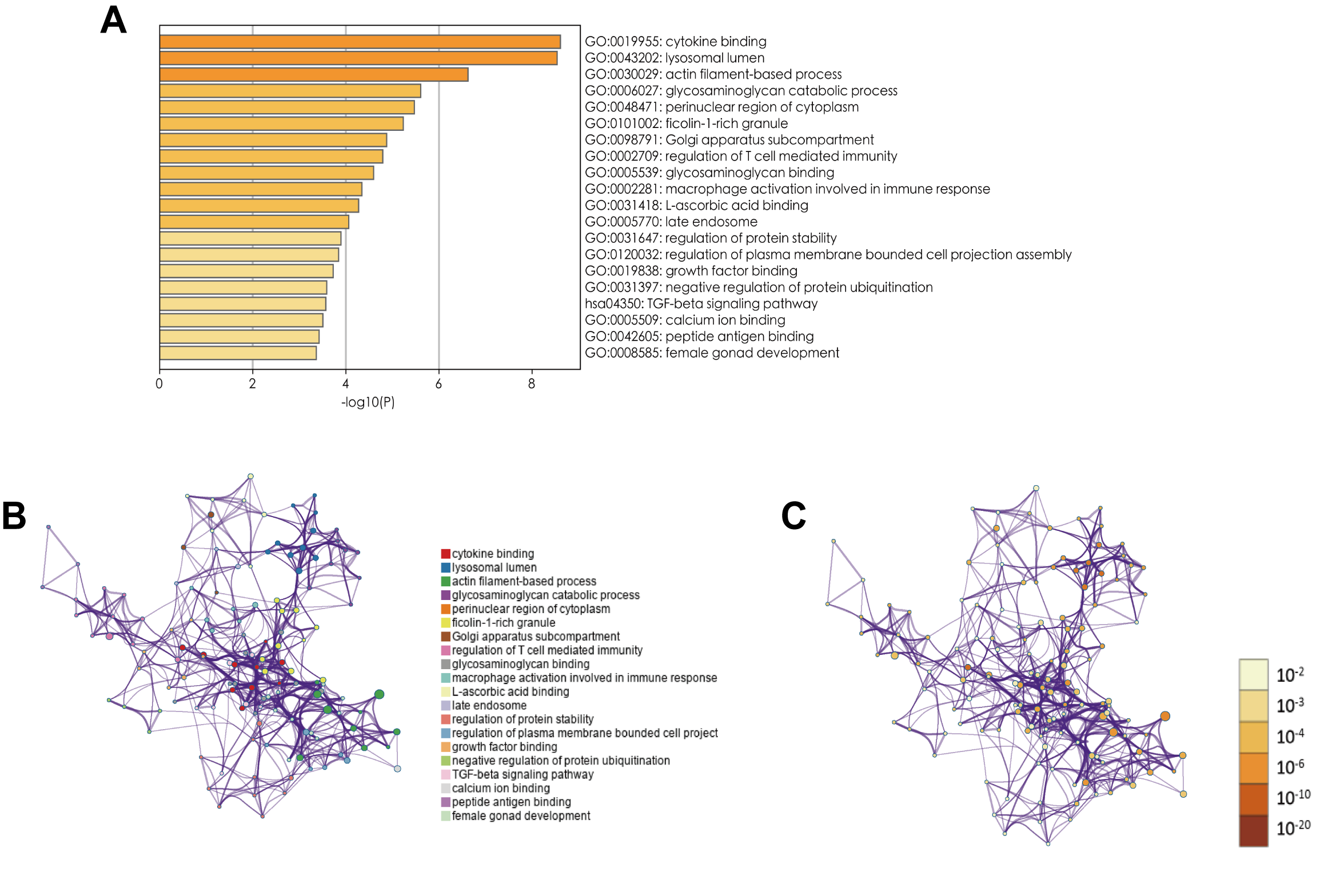


**Supplementary Figure 1 |** Functional annotations enrichment analyses by Metascape. **(A)** Heatmap of enriched terms across input aberrant gene expression profiling, colored by p-values, via the Metascape. **(B)** Network of enriched terms colored by cluster identity using Metascape. **(C)** Network of enriched terms colored by p-value using Metascape.


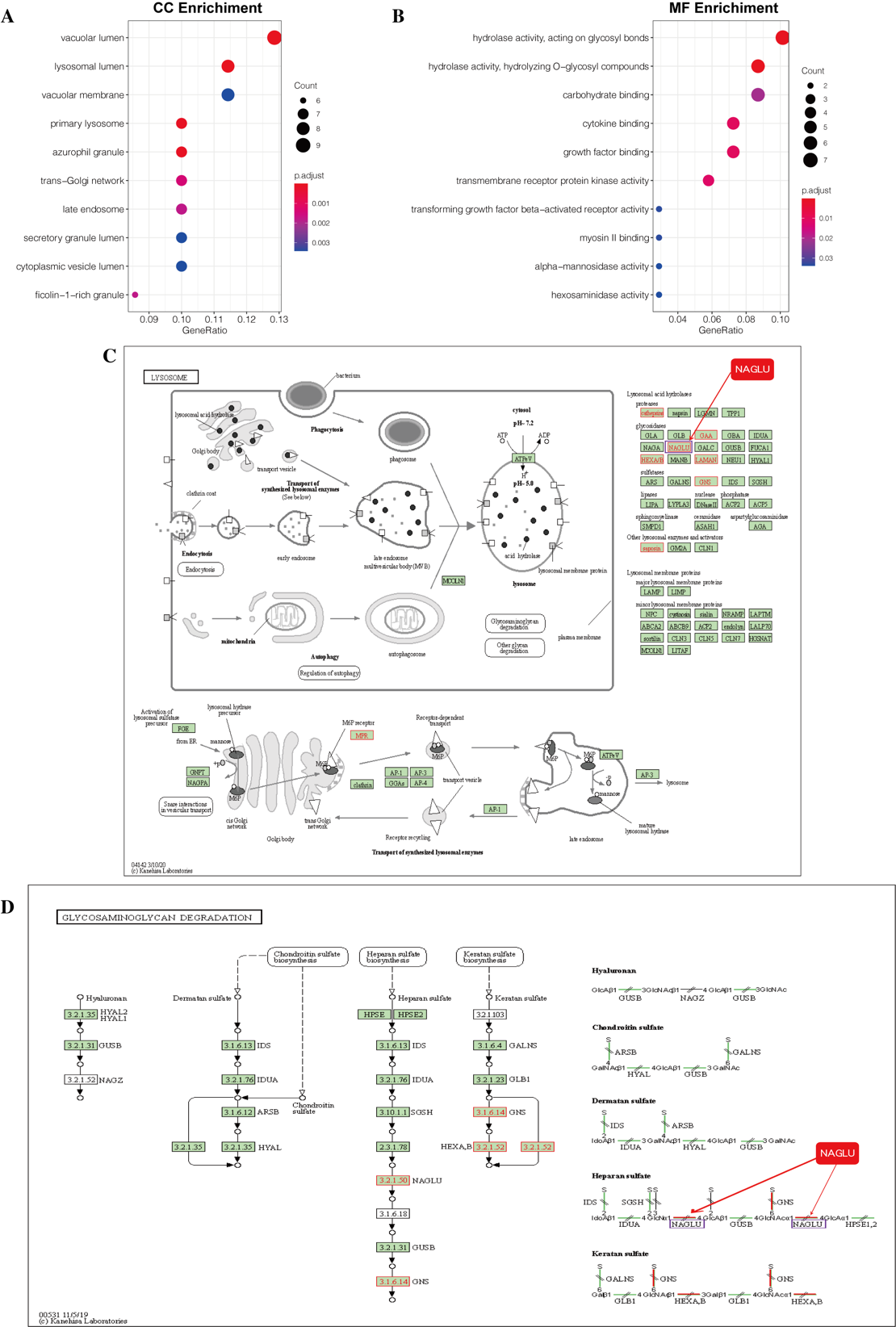


**Supplementary Figure 2 |** Functional annotations enrichment analyses of DRGs in EAS. **(A)** GO CC enrichment of DRGs using R. **(B)** GO MF pathways enrichment of DRGs using R. **(C)** Schematic diagram of lysosome signaling pathway. **(D)** Schematic diagram of GAGs degradation signaling pathway.


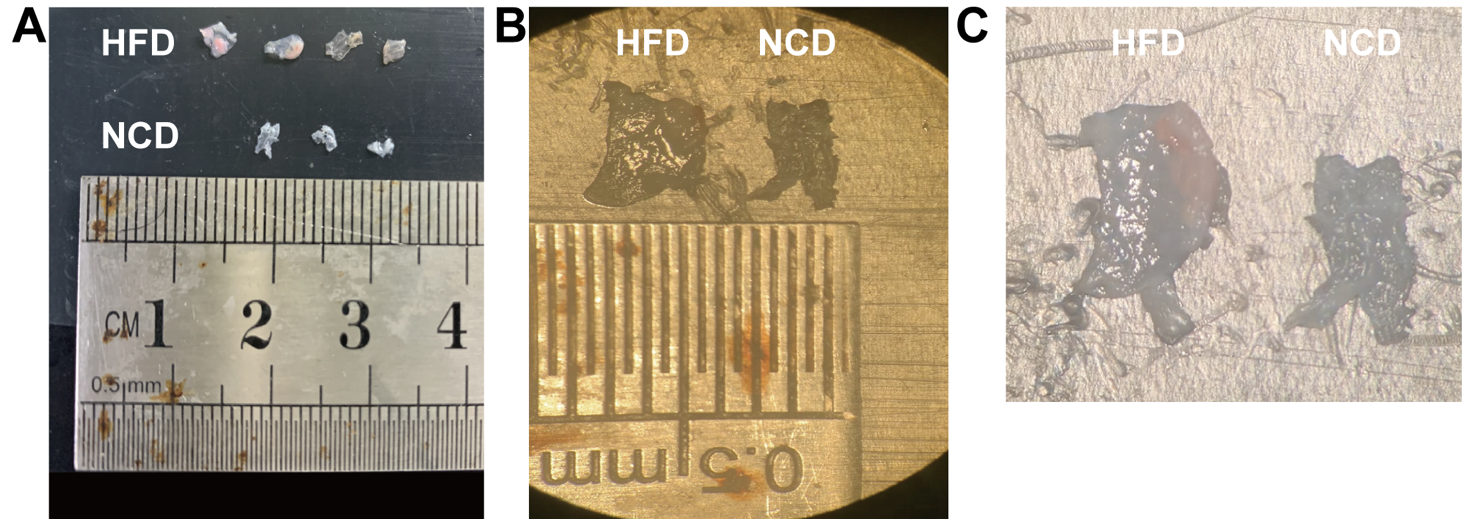


**Supplementary Figure 3 |** Oil red O staining of arterial tissue in *ApoE^-/-^* mice. **(A)** Oil red O staining of arterial tissues of *ApoE^-/-^* mice in HFD group (n=4) and NCD group (n=3). **(B)** and **(C)** Representative images of the oil red O staining of the mice arterial tissues in the HFD group and the NCD group under the microscope (surgical microscope, no scale bar).
